# Benign thyroid nodules respond to a single administration of 0.3mg recombinant human thyrotropin with highly variable volume increase

**DOI:** 10.3389/fendo.2022.1066379

**Published:** 2023-01-06

**Authors:** Panagiotis Bountouris, Georgios K. Markantes, Irene Mamali, Kostas B. Markou, Marina A. Michalaki

**Affiliations:** Division of Endocrinology, Department of Internal Medicine, School of Health Sciences, University of Patras, Patras, Greece

**Keywords:** recombinant human thyrotropin (rhTSH), thyroid volume, thyroid nodule, isoechoic nodule, hypoechoic nodule

## Abstract

**Introduction:**

The nature of thyroid nodules is heterogenous. Most of them are benign and, in the absence of pressure symptoms of adjunct structures, no treatment is needed. Our objective was to investigate the acute effects of a low dose of recombinant human TSH (rhTSH) on the volume of benign thyroid nodules.

**Methods:**

we studied 27 nodules (14 isoechoic and 13 hypoechoic) in 15 (11 women and 4 men; mean age: 51.0 ± 15.9 years) consecutive patients with one to three well-separated asymptomatic benign thyroid nodules. All subjects were euthyroid, with negative thyroid antibodies, and none received levothyroxine. The total thyroid volume and thyroid nodule volume were sonographically determined by two independent examiners (P.B. and M.M.) before, 48 hours and 6 months post intramuscular (IM) administration of 0.3mg rhTSH, and the mean values of the two examiners’ measurements were used; thyroid function tests were obtained at the same time points.

**Results:**

The mean volume of isoechoic nodules increased by 57.3%, of hypoechoic nodules by 46.6% and of the surrounding thyroid parenchyma by 70.4% 48 hours post-rhTSH; mean volumes had returned to baseline levels 6 months later. A large variance in the volume change responses was observed. The relative change in nodule volume (defined as the percent change in nodule volume divided by the percent change in the surrounding parenchyma) from baseline to 48 hours was significantly higher in isoechoic versus hypoechoic nodules (p<0.05).

**Conclusions:**

A single dose of 0.3 mg rhTSH transiently increased the volume of benign thyroid nodules. The increase was more pronounced in isoechoic nodules and had a great variability. Our findings could be useful in the management of benign thyroid nodules, by helping in understanding which nodules would be more responsive to TSH suppression therapy.

## Introduction

Thyroid nodular disease is common in adults, especially in women and the elderly (1). The wider use of thyroid ultrasound as a screening tool has led to increasing rates of thyroid nodule detection, reaching up to 76% of the adult population (2). Thyroid nodules are discrete lesions within the thyroid parenchyma and may be solitary or multiple. More than 90% of thyroid nodules are asymptomatic benign lesions (3, 4). Over time, the size of benign thyroid nodules might increase, decrease, or remain stable, whereas malignant transformation is extremely rare (4).

From a histological perspective, benign thyroid nodules constitute a heterogenous group of lesions, including hyperplastic nodules in multinodular goiters, follicular or Hurthle cell adenomas, nodules in Hashimoto’s or Graves’ disease, colloid nodules, and cysts (colloid, simple, or hemorrhagic) (5, 6). Still, the management of all benign nodules is the same. Levothyroxine (LT4) suppressive therapy was popular in the past but is not recommended in iodine sufficient populations any more due to its uncertain efficacy to reduce the size of thyroid nodules, and to the concerns regarding the adverse effects of iatrogenic hyperthyroidism (7). The rationale for thyrotrophin (TSH) suppression therapy originates from the knowledge that TSH exerts mitogenic effects on follicular thyroid cells *via* binding to its receptor (TSHR) (8). Nonetheless, clinical evidence regarding the response of benign thyroid nodules to TSH is lacking. Recombinant human thyroid-stimulating hormone (rhTSH) became available in the early 1990’s after its purification (9) and, nowadays, it is routinely used in the management of patients with differentiated thyroid cancer (7). Also, it has been evaluated (off-label) as adjuvant to ^131^I treatment of benign nodular goiter due to its ability to enhance thyroid radioactive iodine uptake (10).

The aim of our study was to investigate the acute effect of a low dose of rhTSH on the volume of thyroid nodules in subjects with benign non-toxic nodular thyroid disease.

## Materials and methods

### Study design and participants

We studied 27 benign, non-functioning thyroid nodules. Fifteen (11 women and 4 men; mean age: 51.0 ± 15.9 years, range: 23-77 years old) consecutive patients with one to three well-separated asymptomatic thyroid nodules, otherwise healthy, were recruited between March and June 2020 from the outpatient clinic of the Division of Endocrinology, University Hospital of Patras, Greece. Five of the 11 studied women were postmenopausal. None of the participants had ever received LT4 suppressive therapy for their nodules, and none received medication interfering with thyroid function.

Screening included a complete past medical history and physical examination, thyroid function tests (TFTs) [thyrotropin (TSH), free thyroxine (FT4), triiodothyronine (T3), thyroglobulin (Tg), anti-thyroid peroxidase antibodies (Anti-TPO), anti-thyroglobulin antibodies (Anti-Tg), and calcitonin (CT)], urinary iodine excretion (UIE) in a spot urine sample and fine needle aspiration (FNA) biopsy of the thyroid nodule(s). Inclusion criteria were serum TSH 0.8-4.2 μIU/mL and thyroid hormones within normal range, negative thyroid antibodies, calcitonin <10 pg/mL and benign nodule cytology (category II according to the Bethesda system) (6). Exclusion criteria included known cardiovascular disease, diabetes, or any other serious disease, as well as pregnancy.

Thyroid ultrasound was performed by two independent examiners (P.B. and M.M.) using a 10 MHz linear array transducer (GE Logiq V5 Expert L6-L12RS, GE Healthcare, Chicago, IL, USA). The length (l), width (w), and depth (d) of each nodule and each thyroid lobe were measured on transverse and longitudinal scans, and the nodule echogenicity was assessed. The volume of each thyroid lobe and thyroid nodule were calculated using the modified formula of rotation ellipsoid: 0.479 × l × w × d (11); the mean value of the two examiners’ measurement was used. Total thyroid volume (TV) was the sum of the volume of the two lobes. To study the effects of rhTSH on the nodules and on the rest of the thyroidal tissue separately, we introduced a new variable, the surrounding parenchyma volume (SPV), defined for each participant as the total thyroid volume minus the volume(s) of the nodule(s) present in their thyroid gland.

After screening, eligible participants received an intramuscular (IM) injection of 0.3mg of rhTSH (Thyrogen^®^). This low dose was used to avoid adverse effects, such as excessive thyroid gland swelling or hyperthyroidism. TFTs and thyroid ultrasound were obtained at 48 hours and 6 months after rhTSH administration. For one week after rhTSH administration, daily telephone contacts with the participants were performed to screen for adverse effects. Thyroid nodules were considered significantly responsive to rhTSH if they showed an increase of ≥3mm in maximal diameter and/or ≥50% in volume after the administration of rhTSH; these cut-offs were selected because they represent the minimum changes in size and volume that can be reliably detected by ultrasonography (12).

All the blood samples were centrifuged after collection, and the serum was stored aliquoted at −80°C until analysis. The study protocol was approved by the Ethics Committee of the University Hospital of Patras and all participants provided written informed consent.

### Assays

Serum TSH, T3, FT4, Tg, Anti-TPO, Anti-Tg, and CT were measured by electrochemiluminescence immunoassays - “ECLIA” (Cobas e601, Roche Diagnostics^®^, Mannheim, Germany). The samples were assayed in a single large batch. The intra- and inter-assay precision CV (%) values were 1.1%–3.0% and 3.2%–7.2% for TSH, 1.3%–3.1% and 3.4%–4.5% for T3, 1.1%-4.3% and 2.6%–8.4% for FT4, 2.0–4.8% and 4.0–5.9% for Tg, 4.0%–7.7% and 5.5%–11.7% for Anti-TPO, 1.3-4.9% and 2.1-6.3% for Anti-Tg, and 1.4-1.8% and 1.9-2.3% for CT, respectively. The values of the lower detection limits were 0.005 μIU/mL for TSH, 0.195 ng/mL for T3, 0.0388 ng/dL for FT4, 0.04 ng/mL for Tg, 5.00 IU/mL for Anti-TPO, 10.00 IU/mL for Anti-Tg, and 0.5 pg/mL for CT. UIE was measured using a colorimetric assay based on the Sandell-Kolthoff reaction, according to the method of Dunn et al. (13).

### Statistical analysis

Data were analyzed using IBM SPSS Statistics for Windows, version 27.0 (IBM Corp., Armonk, N.Y., USA). Variables were tested for normality with the Kolmogorov-Smirnov test. Comparisons of TFTs and thyroid/nodule volumes between the different time points were performed using repeated measures ANOVA with a Greenhouse-Geisser correction and with *post-hoc* Bonferroni test (normally distributed variables) and using the Friedman test with posthoc Wilcoxon signed-rank test (non-normally distributed variables). Comparisons of the surrounding parenchyma volume, isoechoic and hypoechoic nodules regarding their volume change between the three time points studied were performed with the Kruskal-Wallis or the Mann-Whitney test, as appropriate. Correlations were estimated by Pearson or Spearman correlation tests, as appropriate. All tests were 2-tailed and a p-value of less than 0.05 was considered significant.

## Results

We studied 27 non-functioning benign thyroid nodules in 15 patients with benign non-toxic nodular thyroid disease. Eight patients had a single thyroid nodule, and seven (5 women and 2 men) had 2-3 nodules. Six of these patients had at least 2 nodules of the same echotexture (**Supplementary Table 1**). All nodules had a cytological diagnosis of benign follicular nodule (Bethesda category II), obtained by FNA. Fourteen of them had isoechoic and 13 hypoechoic sonographic appearance.

The dose of 0.3mg of rhTSH was well tolerated and no serious adverse effects related to thyroid growth or to thyroid hyperfunction were reported by our patients within one week of administration. Three patients reported tachycardia and fatigue in the first 2-3 days post-rhTSH administration, but these symptoms were mild and subsided without treatment.

The mean serum TSH, FT4, T3 and Tg were increased (by 724%, 107%, 34%, and 452%, respectively) 48 hours after the administration of rhTSH compared to baseline, and 6 months later were found to be similar to the baseline values (**Table 1**). The median UIE of the participants in the study was 147.30 mcg/L.

**Table 1:** Thyroid function tests, total thyroid, surrounding parenchyma and nodules’ volumes, and maximum nodule diameter measured before and 48 hours and 6 months after the administration of human recombinant TSH.

	Baseline	48 hours	6 months
Thyroid function tests – all participants (n=15)			
TSH (μIU/mL)	1.50 ± 0.64	12.36 ± 5.15**	1.22 ± 0.69^##^
FT4 (ng/dL)	1.19 ± 0.08	2.46 ± 0.46**	1.46 ± 0.26^##^
T3 (ng/mL)	1.23 ± 0.24	2.53 ± 0.53**	1.10 ± 0.14^##^
Tg (ng/mL)	34.9 (88.73)	192.6 (313.95)**	41.22 (101.18)^#^
Volume parameters			
Total Thyroid Volume – TV (mL)	10.27 (6.24)	15.84 (7.90)**	9.72 (6.10)^##^
Surrounding Parenchyma Volume – SPV (mL)	8.83 ± 2.86	14.71 ± 5.74**	9.30 ± 3.82^##^
All nodules Volume (mL) (n=27)	0.49 (2.13)	1.25 (2.58)**	0.49 (1.82)^#^
Isoechoic nodules Volume (mL) (n=14)	1.10 (2.51)	1.63 (2.91)*	1.19 (2.06)^#^
Hypoechoic nodules Volume (mL) (n=13)	0.35 (0.41)	0.43 (1.03)*	0.39 (0.52)
Maximum nodule diameter (cm)			
All nodules (n=27)	1.57 ± 0.67	1.70 ± 0.71*	1.52 ± 0.63^#^
Isoechoic nodules (n=14)	1.83 ± 0.75	1.99 ± 0.72*	1.79 ± 0.63^#^
Hypoechoic nodules (n=13)	1.29 ± 0.43	1.39 ± 0.56	1.23 ± 0.49

*p<0.05 versus baseline, **p<0.001 versus baseline, ^#^p<0.05 versus 48 hours, ^##^p<0.001 versus 48 hours.

Values are presented as mean ± SD (normally distributed data) or as median (IQR) (non-normally distributed data).

**Figure 1:**
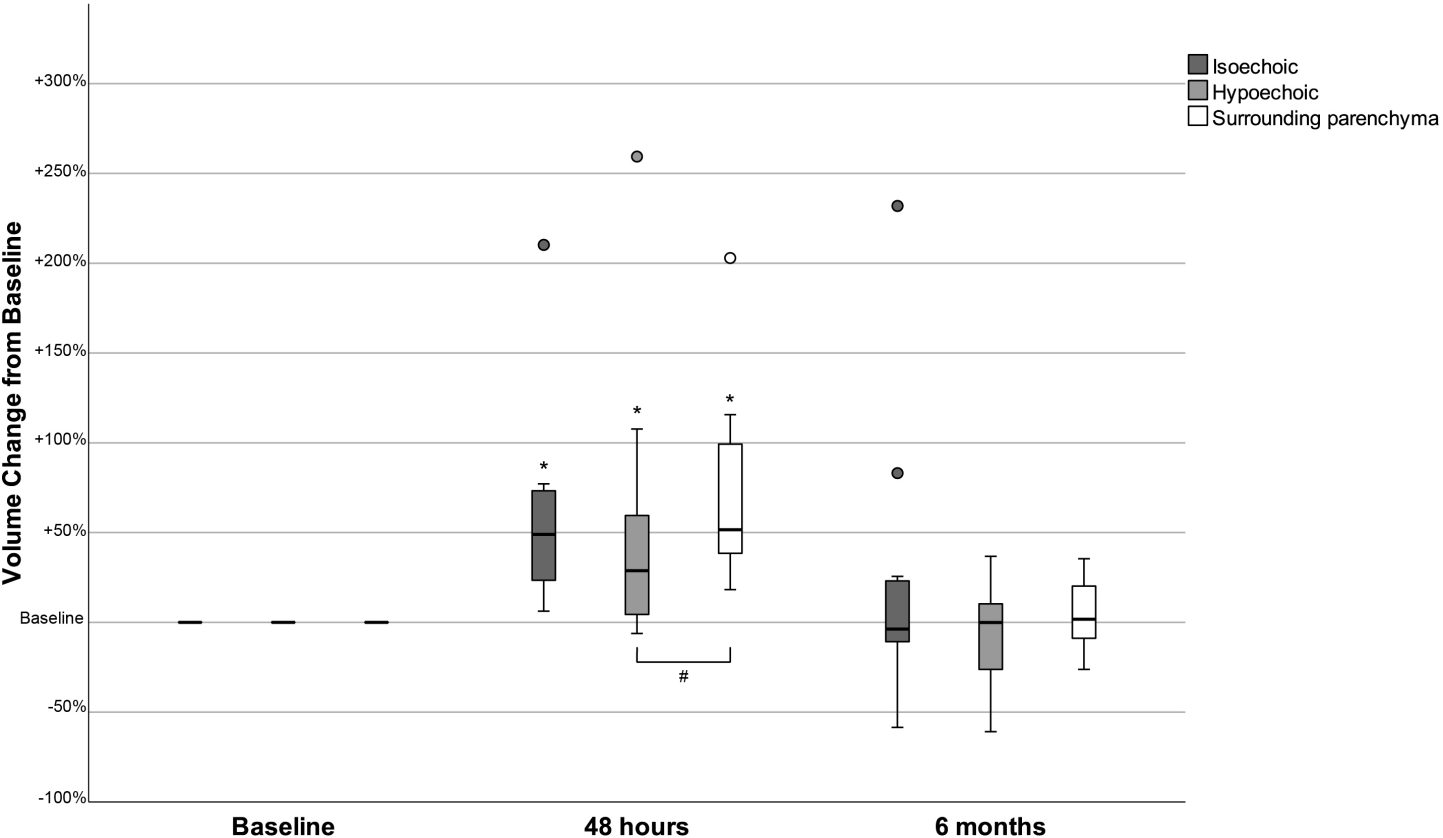
Changes in the volume of isoechoic and hypoechoic nodules and in the surrounding parenchyma volume 48 hours and 6 months after the administration of human recombinant TSH, presented as % change from baseline. *p<0.05 Volume at 48 hours versus baseline (Wilcoxon signed-rank test). p<0.05 Volume change from baseline (Mann-Whitney test).

At baseline, the volume of isoechoic nodules was larger than that of hypoechoic [median (IQR): 1.10 (2.51) mL vs 0.35 (0.41) mL, p=0.026]. The mean TV, SPV and the mean nodules volume (either when all nodules were studied together or when isoechoic and hypoechoic were analyzed separately) were significantly increased 48 hours post-rhTSH and had returned to baseline levels 6 months later (**Table 1**; **Figure 1**). The mean maximum nodule diameter increased significantly at 48 hours and decreased at 6 months in isoechoic nodules; in hypoechoic nodules these differences did not reach statistical significance (**Table 1**). The mean increase of SPV observed at 48 hours post-rhTSH was 70.4% and had no statistically significant difference from that of isoechoic nodules (57.3%), whereas it was significantly greater from that of hypoechoic nodules (46.6%, p=0.029). Besides, the relative change in nodule volume (defined as the percent change in nodule volume divided by the percent change in the SPV) from baseline to 48 hours was significantly higher in isoechoic versus hypoechoic nodules (0.88 vs 0.31, p<0.05, **Supplementary Table 2**).

Twelve of the 27 studied nodules were found to be significantly responsive to rhTSH, meaning that their maximal diameter increased by ≥3mm and/or their volume increased by ≥50% at 48 hours post-rhTSH. The proportions of the isoechoic and hypoechoic nodules that responded significantly to rhTSH were 50% (7/14) and 38.5% (5/13), respectively (p=ns). The responsive and non-responsive nodules did not differ significantly regarding their baseline volume or maximum diameter.

**Figure 2:**
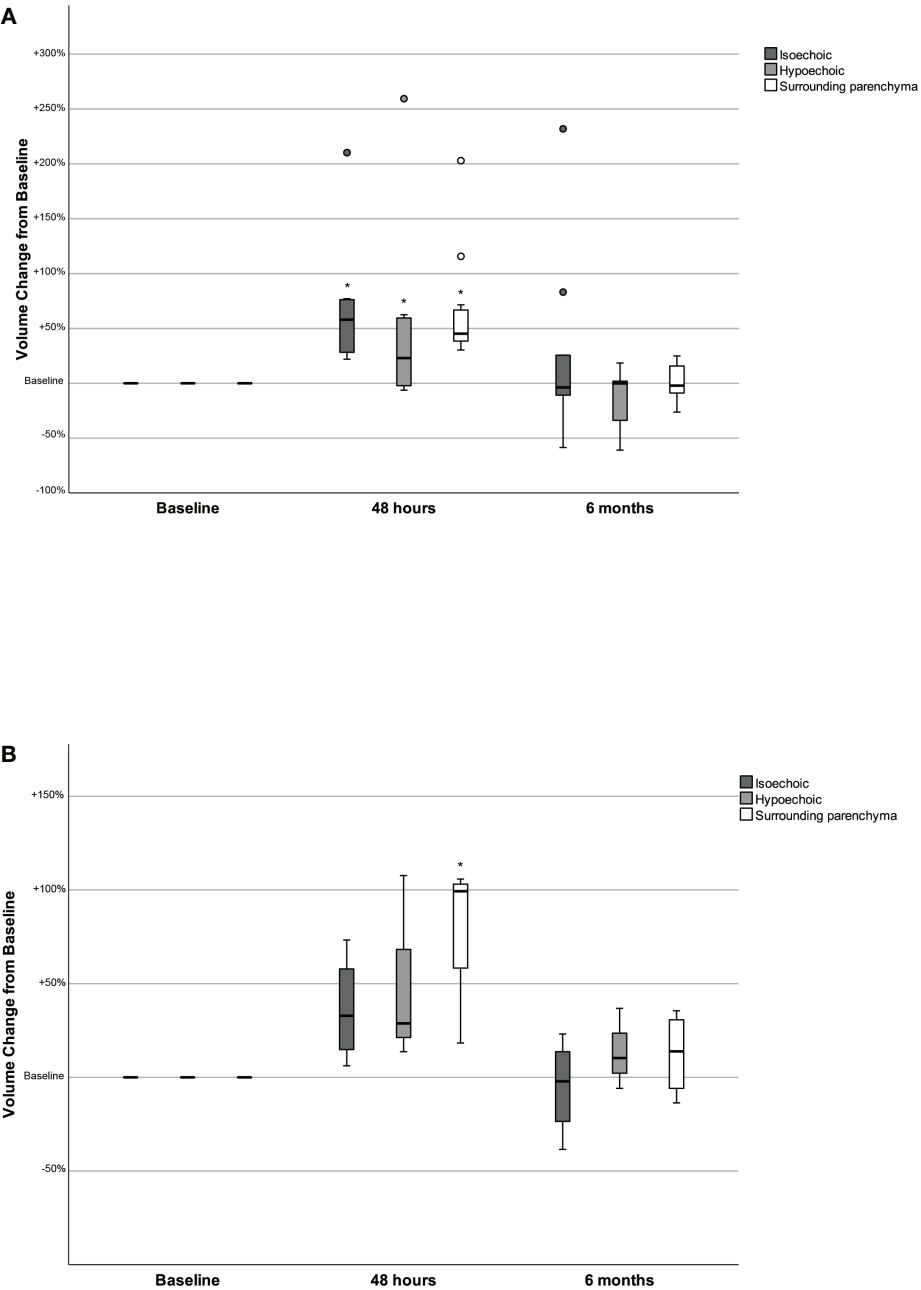
Changes in the volume of isoechoic and hypoechoic nodules and in the surrounding parenchyma volume 48 hours and 6 months after the administration of human recombinant TSH, presented as % change from baseline, in women **(2A)** and men **(2B)**. *p<0.05 Volume at 48 hours versus baseline (Wilcoxon signed-rank test).

When women and men were studied separately, the observed alterations in TFTs, TV, SPV, nodules volume and maximum nodule diameter in women were identical to those of the whole patient cohort (**Table 2**; **Figure 2A**). In men, the changes in TSH, T3, Tg, mean isoechoic and hypoechoic nodule volume and mean maximum nodule diameter were not statistically significant (**Table 2**; **Figure 2B**), possibly due to the small number of male participants.

**Table 2:** Thyroid function tests, total thyroid, surrounding parenchyma and nodules’ volumes, and maximum nodule diameter measured before and 48 hours and 6 months after the administration of human recombinant TSH, in male and female participants.

	Baseline	48 hours	6 months
Thyroid function tests – women (n=11)/men (n=4)			
TSH (μIU/mL)	1.60 ± 0.68/1.24 ± 0.46	13.43 ± 4.68**/8.41 ± 5.71	1.18 ± 0.79^##^/1.36 ± 0.33
FT4 (ng/dL)	1.18 ± 0.08/1.22 ± 0.10	2.39 ± 0.44**/2.72 ± 0.51*	1.47 ± 0.29^##^/1.42 ± 0.22
T3 (ng/mL)	1.23 ± 0.22/1.25 ± 0.32	2.49 ± 0.46**/2.65 ± 0.87	1.12 ± 0.14^##^/1.05 ± 0.13
Tg (ng/mL)	34.4 (94.96)/99.93 (235.60)	142.0 (231.81)**/393.30 (303.70)	41.22 (97.95)^#^/72.89 (142.21)
Volume parameters – women (n=11)/men (n=4)			
Total Thyroid Volume – TV (mL)	8.33 (3.79)/16.18 (7.65)	12.85 (4.20)**/26.60 (16.42)*	9.13 (3.90)^##^/16.58 (10.20)^#^
Surrounding Parenchyma Volume – SPV (mL)	7.54 ± 1.87/12.39 ± 1.90	11.98 ± 2.32**/22.21 ± 5.82*	7.60 ± 1.92^##^/13.98 ± 4.02^#^
All nodules Volume (mL) (n=20/7)	0.45 (0.62)/2.86 (4.12)	0.52 (1.09)**/4.07 (4.45)*	0.44 (0.60)^#^/2.98 (3.62)^#^
Isoechoic nodules Volume (mL) (n=10/4)	0.77 (2.12)/3.87 (5.41)	1.38 (2.51)*/ 5.04 (5.20)	0.57 (1.78)^#^/3.72 (2.92)
Hypoechoic nodules Volume (mL) (n=10/3)	0.34 (0.28)/0.75 (–)	0.40 (0.43)*/ 1.56 (-)	0.32 (0.40)/0.83 (-)
Maximum nodule diameter (cm) – women (n=11)/men (n=4)			
All nodules (n=20/7)	1.34 ± 0.50/2.20 ± 0.70	1.47 ± 0.56*/ 2.36 ± 0.71	1.30 ± 0.49^#^/2.16 ± 0.51
Isoechoic nodules (n=10/4)	1.56 ± 0.60/2.50 ± 0.72	1.75 ± 0.60*/ 2.60 ± 0.68	1.54 ± 0.52^#^/2.41 ± 0.42
Hypoechoic nodules (n=10/3)	1.13 ± 0.28/1.81 ± 0.51	1.19 ± 0.35/ 2.04 ± 0.73	1.05 ± 0.34/1.83 ± 0.48

*p<0.05 versus baseline, **p<0.001 versus baseline, ^#^p<0.05 versus 48 hours, ^##^p<0.001 versus 48 hours.

Values are presented as mean ± SD (normally distributed data) or as median (IQR) (non-normally distributed data). /, seperates the values corresponding to women and men, respectively, and -, is used to denote that a median value is not available as there were only 3 hypoechoic nodules in men.

**Figure 3:**
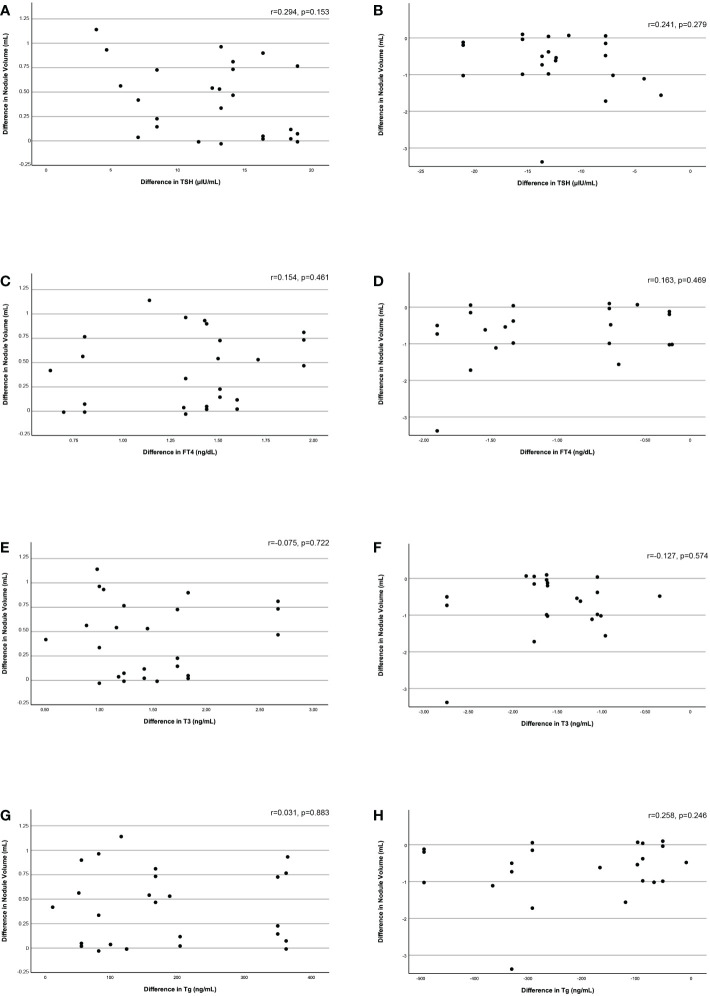
Correlation of the difference in nodule volume between baseline and 48 hours and between 48 hours and 6 months with the respective difference in serum TSH **(3A, 3B)**, FT4 **(3C, 3D)**, T3 **(3E, 3F)**, and Tg **(3G, 3H)** levels.

There was a strong correlation between the difference in hypoechoic nodule volume and the difference in SPV (Spearman’s rho=0.692, p=0.009 - 48 hours vs baseline, Spearman’s rho=0.770, p=0.002 – 6 months vs 48 hours), while isoechoic nodule volume differences were not correlated with SPV alterations. The differences in nodule volume between the three time points studied were not correlated with the respective differences in TSH, FT4, T3, or Tg (**Figure 3**).

## Discussion

To our knowledge, this is the first study investigating the effects of rhTSH on the volume of benign thyroid nodules in patients with non-toxic nodular thyroid disease. We examined otherwise healthy, middle-aged patients living in an iodine sufficient area. We found that the volume of the nodules and the surrounding thyroid parenchyma were significantly increased 48 hours post a single IM administration of 0.3 mg of rhTSH and had returned to baseline values six months later. Notably, the magnitude of the volume increase differed between isoechoic nodules, hypoechoic nodules, and the surrounding thyroid parenchyma.

The process of goitrogenesis is not well understood (14). Iodine deficiency is an established cause of goiter but is not likely in our patients. Greece has become an iodine sufficient country since the 1990’s (15, 16), and the mean UIE of our subjects was well above the lower limit of normal set by the World Health Organization (WHO) (17). However, since our patients were middle-aged, we cannot exclude the possibility that some of them might have had inadequate iodine intake in the past. Furthermore, none of our subjects had thyroid autoimmunity, so the cause of their goiter was not apparent.

Several studies have examined the effects of rhTSH on thyroid RAI uptake, thyroid function, and thyroid volume in euthyroid volunteers with and without nodular goiter, with doses ranging from 0.01 to 0.9 mg (18–20). Two of them reported on changes in thyroid volume in euthyroid healthy volunteers with normal sonographic thyroid morphology, after rhTSH administration. In the first, a single IM injection of 0.9mg rhTSH transiently enlarged the thyroid gland, with the peak of the volume increase (35.5%) occurring at 48 hours (20). In the second, the investigators reported a slight (approximately 10%) but significant increase in thyroid volume 48 hours after the IM administration of 0.1mg of rhTSH, which had returned to baseline values 4 days post rhTSH administration (19). In another study, Nielsen et al. investigated the effect of a single IM administration of 0.3 mg rhTSH on the volume of benign nontoxic multinodular goiters and found a maximum volume increase of 24% at 48 hours. The goiter volume had returned to baseline values after a week and remained at this level on the 28^th^ day post-rhTSH (21); importantly, only the total thyroid volume and not each nodule’s volume was assessed in this study. In our study, we observed a mean increase of 70% in TV from baseline to 48 hours, which was greater compared to previous studies (19–21), a finding that we cannot easily explain and could be attributed to differences between the studied populations and in the methodology used in each study. Regarding thyroid function, it seems that a dose-depended correlation between rhTSH administration and the increase in thyroid hormones exists for doses up to 0.3mg; thereafter, the maximal stimulation has probably been achieved, and the response regarding the serum levels of thyroid hormones is similar for 0.3 and 0.9mg of rhTSH (18, 22). It is not known how rhTSH administration leads to the acute enlargement of the thyroid gland. It has been hypothesized that an exaggerated vascular response and interstitial fluid accumulation could be implicated (20, 21). However, the possibility of hypertrophy or proliferation of follicular cells and enlargement of the follicles cannot be excluded. *In vitro* studies, in cultures of normal or cancerous animal or human thyroid cell lines, have shown a mitogenic effect of bovine TSH in cooperation with insulin/IGF-1 on follicular cells (8, 23, 24). In primary cultures of normal thyroid cells and adenomatous goiter cells treatment with TSH stimulated cell proliferation, as shown by decreased doubling times (106 to 76 hours) (23) and increased DNA synthesis (24). However, other studies do not support the notion that TSH stimulates proliferation of normal human thyrocytes in culture (25, 26). Besides, in cultures of thyroid papillary and follicular cancer cells a biphasic response to TSH has been reported: low concentrations of TSH stimulated invasion and growth of cancerous cells, whereas these were inhibited by high TSH concentrations (27). These discrepancies might arise from the different types of thyroid cells used (animal/human, normal/goiter/cancer), the different culture conditions, and the different doses of rhTSH used in the various studies. *In vivo*, the hypothalamus-pituitary-thyroid axis is a system characterized by dynamic compensation, meaning that the regulated variable (thyroid hormones) controls the functional mass dynamics of its regulating tissues (pituitary and thyroid glands) (28). This explains the delays in TSH normalization after correction of hypo- and hyperthyroidism (hysteresis). In these disease states, it normally takes weeks to months for the abnormal levels of thyroid hormones or TSH to alter the pituitary or thyroid cell mass (29); our study shows that even acute, significant elevations of TSH might stimulate thyroid cell growth.

Notably, in our study, the magnitude of the response to 0.3 mg of rhTSH was different between isoechoic nodules, hypoechoic nodules, and the surrounding thyroid parenchyma, showing considerable variability. In particular, the volume change in hypoechoic nodules was smaller than that of the isoechoic nodules. Furthermore, the mean maximum diameter of isoechoic nodules increased significantly post rhTSH administration, while in hypoechoic nodules it did not. We do not know with certainty what determines the echogenicity of a nodule. In a few studies, it seems that marked fibrosis (>30%) contributes to hypoechoic sonographic appearance (30, 31). We might hypothesize that hypoechoic nodules respond less to rhTSH due to increased fibrosis compared to isoechoic nodules or to the SPV. Nodular thyroid disease results from follicular cell hyperplasia and multiclonal cell proliferation (14). The follicles of thyroid nodules are heterogeneous regarding their morphology, growth pattern, mitotic activity, and function, exactly like the “normal”, non-nodular thyroid tissue (32, 33). In this context, it is not surprising that we observed striking differences (large variance) in the volume change responses of the studied nodules to rhTSH. Even nodules of the same echotexture and within the same thyroid gland demonstrated marked inconsistency regarding their volume response to rhTSH.

Our study has several limitations. First, we do not know when the maximum of volume response occurred and when the nodules returned to their baseline size, since we only took measurements at 48 hours and at 6 months following rhTSH administration. Besides, thyroid scintigraphy was not performed to rule-out hyperfunctioning nodules; however, we recruited subjects with TSH ≥0.8 μIU/mL, which makes the possibility of toxic nodular disease unlikely (34). Lastly, we only studied the acute effect of rhTSH on thyroid nodules and our results cannot be extrapolated to other conditions (such as chronically elevated TSH).

In conclusion, a single dose of 0.3 mg rhTSH transiently increased the volume of benign thyroid nodules, but their response demonstrated a great variability and was more pronounced in isoechoic nodules. In clinical practice all benign thyroid nodules are managed as one category, when in fact there is great heterogeneity between them. Our findings could be helpful in the management of benign thyroid nodules. In fact, we aim to conduct an interventional study in which LT4 suppressive therapy will be administered to patients with nodules that significantly increased in size post-rhTSH. More clinical studies are needed to confirm our conclusions.

## Data availability statement

The raw data supporting the conclusions of this article will be made available by the authors, without undue reservation.

## Ethics statement

The studies involving human participants were reviewed and approved by Ethics Committee of the University Hospital of Patras. The patients/participants provided their written informed consent to participate in this study.

## Author contributions

PB: data collection, statistical analysis, writing of the manuscript. GM: statistical analysis, writing of the manuscript. IM: hormone measurements. KM: study design, critical revision of the manuscript. MM: study design, data collection, writing of the manuscript. All authors contributed to the article and approved the submitted version.

## Supplementary material

Supplementary Table 1 Summary of patients’ characteristics.

Supplementary Table 2 Relative change in nodules volume (defined as the percent change in nodule volume divided by the percent change in the SPV) from baseline to 48 hours and from 48 hours to 6 months (I, Isoechoic; H, Hypoechoic).
