# Supplementary material for: Benign thyroid nodules respond to a single administration of 0.3mg recombinant human thyrotropin with highly variable volume increase
Source: Front Endocrinol (Lausanne). 2023 Jan 6;13:1066379. doi: 10.3389/fendo.2022.1066379 (PMC9875562; doi:10.3389/fendo.2022.1066379)
Supplement: Supplementary Table 1 — Summary of patients’ characteristics. [file Table_1.doc]

**Supplementary Table 1:** Summary of patients’ characteristics.

| Patient | Age | Sex | Number of nodules | Nodule Type  I: Isoechoic  H: Hypoechoic | Nodule Volume (mL)  Baseline, 48 hours, 6 months | TSH (μIU/mL) Baseline, 48 hours, 6 months | FT4 (ng/dL) Baseline, 48 hours, 6 months | T3 (ng/mL) Baseline, 48 hours, 6 months | Tg (ng/mL) Baseline, 48 hours, 6 months | UIE (mcg/L) |
| --- | --- | --- | --- | --- | --- | --- | --- | --- | --- | --- |
| 1 | 72 | F | 3 | I  I  I | 0.24, 0.38, 0.44 / 3.14, 3.86, 2.14 / 0.30, 0.52, 0.37 | 1.50, 9.74, 1.75 | 1.26, 2.77, 1.12 | 1.16, 2.89, 1.13 | 154.5, >500, 202.7 | 210.0 |
| 2 | 35 | F | 1 | I | 0.78, 1.34, 0.33 | 1.84, 7.36, 0.06 | 1.08, 1.87, 1.73 | 1.21, 2.09, 1.08 | 35.7, 84.8, 14.5 | 75.0 |
| 3 | 67 | F | 1 | I | 0.75, 1.17, 0.69 | 2.47, 9.31, 1.31 | 1.20, 1.82, 1.22 | 1.12, 1.62, 1.27 | 4.6, 14.8, 4.6 | 89.12 |
| 4 | 77 | M | 3 | I  I  H | 7.54, 8.0, 4.63 / 1.0, 1.7, 1.23 / 0.75, 1.56, 0.83 | 1.04, 14.96, 1.02 | 1.28, 3.23, 1.33 | 0.99, 3.66, 0.92 | 336.6, >500, 164.2 | 147.30 |
| 5 | 42 | M | 1 | I | 4.88, 6.02, 4.45 | 0.77, 4.48, 1.67 | 1.07, 2.21, 1.67 | 1.15, 2.13, 1.17 | 84.1, 196.3, 72.9 | 210.30 |
| 6 | 65 | F | 3 | I  H  H | 2.41, 3.17, 2.15 / 0.22, 0.21, 0.09 / 0.20, 0.28, 0.08 | 2.70, 21.44, 0.12 | 1.14, 1.94, 1.78 | 1.39, 2.62, 1.01 | 269.0, >500, 127.5 | 280.79 |
| 7 | 46 | F | 1 | I | 2.41, 2.94, 2.32 | 1.90, 14.81, 2.14 | 1.26, 2.97, 1.43 | 1.09, 2.54, 1.30 | 27.9, 212.4, 41.2 | 101.45 |
| 8 | 23 | F | 1 | H | 0.44, 0.43, 0.50 | 2.23, 13.58, 2.14 | 1.14, 1.83, 1.43 | 1.61, 3.15, 1.30 | 21.6, 142.0, 41.2 | 228.86 |
| 9 | 40 | F | 1 | H | 0.33, 0.36, 0.39 | 0.59, 7.42, - | 1.14, 2.46, - | 1.45, 2.63, - | 31.9, 128.2, - | 95.49 |
| 10 | 53 | F | 3 | H  H  I | 0.35, 1.25, 0.26 / 0.06, 0.08, 0.04 / 0.06, 0.11, 0.21 | 1.09, 17.25, 1.48 | 1.09, 2.53, 1.92 | 0.89, 2.72, 1.10 | 2.4, 55.4, 1.4 | 60.53 |
| 11 | 54 | M | 2 | I  H | 2.85, 4.07, 2.98 / 0.28, 0.32, 0.38 | 1.84, -, - | 1.21, -, - | 1.72, -, - | 115.8, -, - | 187.65 |
| 12 | 60 | F | 3 | I  H  I | 0.46, 1.42, 0.44 / 0.48, 0.45, 0.49 / 1.19, 1.53, 1.15 | 1.16, 14.19, 0.85 | 1.16, 2.49, 1.16 | 1.0, 2.0, 0.95 | 34.4, 113.1, 21.8 | 97.52 |
| 13 | 58 | M | 1 | H | 3.24, 4.17, 3.06 | 1.30, 5.78, 1.39 | 1.30, 2.73, 1.27 | 1.13, 2.17, 1.06 | 33.1, 393.3, 22.0 | 174.33 |
| 14 | 28 | F | 2 | H  H | 0.49, 0.51, 0.49 / 0.20, 0.31, 0.20 | 1.06, 19.30, - | 1.17, 2.77, - | 1.47, 2.89, - | 116.6, 316.6 | 59.88 |
| 15 | 45 | F | 1 | H | 0.87, 1.41, 0.87 | 1.00, 13.37, 0.74 | 1.34, 2.84, 1.45 | 1.08, 2.24, 0.96 | 34.9, 188.9, 87.5 | 199.30 |
