# Supplementary material for: Benign thyroid nodules respond to a single administration of 0.3mg recombinant human thyrotropin with highly variable volume increase
Source: Front Endocrinol (Lausanne). 2023 Jan 6;13:1066379. doi: 10.3389/fendo.2022.1066379 (PMC9875562; doi:10.3389/fendo.2022.1066379)
Supplement: Supplementary Table 2 — Relative change in nodules volume (defined as the percent change in nodule volume divided by the percent change in the SPV) from baseline to 48 hours and from 48 hours to 6 months (I, Isoechoic; H, Hypoechoic). [file Table_2.docx]

**Supplementary Table 2:** Relative change in nodules volume (defined as the percent change in nodule volume divided by the percent change in the SPV) from baseline to 48 hours and from 48 hours to 6 months (I: Isoechoic, H: Hypoechoic).

|  | Nodule Type | Relative Volume Increase Baseline to 48 hours | Relative Volume Decrease 48 hours to 6 months |
| --- | --- | --- | --- |
| 1 | I | 0.84 | -0.43 |
| 2 | I | 0.32 | 1.34 |
| 3 | I | 1.07 | 0.87 |
| 4 | I | 1.16 | 1.75 |
| 5 | I | 1.08 | 1.80 |
| 6 | I | 0.06 | 1.24 |
| 7 | I | 0.69 | 0.85 |
| 8 | H | 1.02 | 1.37 |
| 9 | I | 0.23 | 0.70 |
| 10 | I | 0.70 | 0.66 |
| 11 | H | -0.09 | 1.15 |
| 12 | H | 0.79 | 1.45 |
| 13 | I | 0.55 | 0.58 |
| 14 | H | -0.06 | -0.46 |
| 15 | H | 0.29 | -0.22 |
| 16 | H | 6.90 | 2.44 |
| 17 | H | 0.93 | 1.56 |
| 18 | I | 2.05 | -2.69 |
| 19 | I | 2.32 | 0.99 |
| 20 | H | 0.75 | -0.75 |
| 21 | I | 6.91 | 12.60 |
| 22 | H | -0.20 | -1.60 |
| 23 | I | 0.93 | 4.55 |
| 24 | H | 0.29 | 0.55 |
| 25 | H | 0.04 | 0.10 |
| 26 | H | 0.51 | 0.88 |
| 27 | H | 0.31 | 0.59 |
|  |  |  |  |
| Isoechoic Median |  | 0.88 | 0.93 |
| Hypoechoic Median |  | 0.31 | 0.59 |
| p (Isoechoic vs Hypoechoic) |  | 0.048 | 0.302 |
